# Tuberculosis in Mammals and in Birds

**Published:** 1899-11-18

**Authors:** 


### TUBERCULOSIS IN MAMMALS AND IN BIRDS.

The observations made by Dr. Woods Hutchinson[1] upon the local distribution of tubercle in various species of animals are of very great interest, not that they help us much in regard to the etiology of the disease, but rather because they tend to show that some of the cut and dried notions which are prevalent on the subject may not be nearly so true as they are often thought to be. It would appear that in all mammals the lung is the point of severest attack, and its lesions the usual cause of death. But whether it is the earliest to be affected or whether the infection enters by the lung is another matter, on which no light is thrown. The distribution of tubercle in birds is strikingly different. Instead of the lungs being the commonest site of attack they are one of the rarest among the great viscera, and their lesions are very seldom the cause of death. The chief seat of the disease among birds lies in the abdominal viscera; first and most severely in the liver, next in the spleen, and third in the wall of the intestine and in the mesenteric nodes. As showing how great is the doubt which still hangs over the question of the track by which tuberculosis infection reaches the viscera in which it first becomes established, Dr. Hutchinson asks: If the tremendous susceptibility of the lungs in mammals is to be accounted for by their being the port of entry for aerial infection, how are we to explain the almost equally striking immunity of the bird's lung?—to say nothing of the well-known experimental fact that if a guinea-pig or rabbit be inoculated in the flank or abdomen he usually dies of pulmonary tuberculosis, and this, too, in spite of the fact that the guinea-pig is extremely hard to infect, even by coating his cage bottom, or mixing his food with dried sputum, and that according to fanciers he is almost exempt from tuberculosis. The air passages of the bird are certainly no better protected against dust contagion than those of the mammal; indeed, if anything rather less so. What is suggested by these observations is that different classes of animals are specially vulnerable in different organs, that in mammals the lungs are the weak spot, and that by whatever channel the bacilli are introduced they find in the lungs the most favourable soil for growth. Dr. Woods Hutchinson says that, according to Professor MacFadyean, if a series of rabbits and a series of guinea-pigs be inoculated in the same part of the flank from the same culture, in the former the kidney will be affected and the spleen exempt, while in the latter the spleen will be attacked and the kidney will escape. Nor is it to tuberculosis alone that the lungs of mammals are so vulnerable. "In any bills of human mortality the three highest causes of death are almost invariably diseases of the lung, . . . and the same disproportionate fatality is found in the lower animals." In birds, on the other hand, while tubercle is rare in the lung, so are other diseases. "Nearly all of the 'plagues' and fatal diseases of birds attack only the abdominal viscera." It is to be noted that these interesting observations do not touch the question of infection by dust. If infection in the form of dust enters the mouth or the nose it is just as likely—nay, it is more likely—to reach the lungs by way of the lymphatics and the blood stream as by way of the bronchial tubes. The fact of the infection being introduced by means of dried sputum is by no means invalidated. What these observations do tend to upset is the somewhat crude notion that an infection which goes in with the air must arrive at the seat of its development by way of the air passages, which by no means follows. And so we again come back to the importance of predisposition as against that of direct infection. If the reason why tubercle attacks the lungs is to be found in a special vulnerability of mammalian lung tissue it is by removing the conditions of life by which this vulnerability is increased, and not merely by guarding against a few of the more common ways in which the bacillus obtains an entry into the body, that the disease is to be prevented.

[1] Brit. Med. Jour., Nov. 11.

